# Mechanisms of Microplastic Effects on Carbon and Nitrogen Cycling in Aquatic and Terrestrial Ecosystems

**DOI:** 10.3390/toxics14070551

**Published:** 2026-06-24

**Authors:** Xintong Zhang, Yuxiao Chen, Chia Min Ho, Weiying Feng, Xuezheng Yu

**Affiliations:** 1 Shi Jia College, Beihang University, Beijing 100191, China; 25375215@buaa.edu.cn; 2 School of Mechanical Engineering and Automation, Beihang University, Beijing 100191, China; yuxiaozhizuo@buaa.edu.cn; 3 School of Materials Science and Engineering, Beihang University, Beijing 100191, China; hochiamin@buaa.edu.cn (C.M.H.); yuxuezheng@buaa.edu.cn (X.Y.)

**Keywords:** microplastics, diversity, carbon cycle, nitrogen cycle, greenhouse

## Abstract

An emerging environmental pollutant, microplastics have garnered global attention due to their widespread presence in soil and aquatic ecosystems. Early research primarily treated microplastics as single pollutants, focusing on their individual toxic effects. However, microplastics in the environment exist as a complex mixture, comprising various polymer types, sizes, shapes, and aging states. This diversity influences how microplastics regulate ecosystem carbon and nitrogen cycles and intervene through pathways such as direct carbon input, physical disturbance, microbial community restructuring, and coupled effects. This paper systematically reviews the characteristics of microplastic diversity and its mechanisms influencing carbon and nitrogen cycles: the chemical structure of polymers determines bioavailability and degradation rate, with biodegradable plastics altering carbon and nitrogen transformations more significantly than conventional plastics; microplastics of different sizes affect nitrogen transformation dynamics by modulating specific surface area and microbial colonization, with small-sized biodegradable microplastics particularly inhibiting plant nitrogen uptake; aging modifies surface properties and dissolved organic carbon release, thereby enhancing their role in promoting greenhouse gas emissions. Existing studies are largely confined to short-term laboratory simulations, leaving a gap in understanding the comprehensive effects of microplastic diversity under long-term, field conditions. Future research should focus on standardized methods and long-term experiments with multi-factor coupling to provide a scientific basis for ecological risk assessment of microplastic pollution.

## 1. Introduction

While the widespread use of plastic products has brought tremendous convenience to human society, it has also led to increasingly severe environmental pollution problems. It is estimated that millions of tons of plastic waste enter terrestrial and aquatic ecosystems globally each year, gradually degrading into microplastics (particles smaller than 5 mm) and nanoplastics (particles smaller than 1 μm) under the influence of physical fragmentation, ultraviolet radiation, and biological processes [1]. The widespread presence of microplastics and their potential risks to biological health have attracted widespread attention. However, their ecological and environmental effects on a larger scale, particularly their impact on global element cycles, have only recently begun to be systematically studied.

The carbon and nitrogen cycles are the most fundamental elemental cycling processes in ecosystems, directly influencing soil fertility, ecosystem productivity, and global climate change. Microorganisms are the core drivers of carbon and nitrogen transformation, and microplastics intervene in these microorganism-driven elemental cycles through multiple pathways: microplastics themselves, as carbon-rich substrates, can directly participate in the carbon cycle; changes in the physical structure of soil and water bodies caused by microplastics indirectly regulate the environmental conditions for elemental transformation [2]. These effects are not a simple summation of individual pollutants but rather complex processes determined by the multidimensional properties of microplastics, including polymer type, size, morphology, degree of degradation, and associated chemical contaminants.

Early studies often treated microplastics as homogeneous pollutants, focusing on the effects of specific polymers (such as polyethylene and polystyrene) in short-term experiments. However, microplastics in the environment constitute a highly heterogeneous mixture. This review does not aim to establish a single definitive model for the effects of microplastics on carbon and nitrogen cycles. Instead, it synthesizes current evidence, identifies recurring mechanistic patterns, and clarifies major sources of uncertainty. Specifically, we first define the main dimensions of microplastic diversity and then examine how these dimensions influence carbon input and mineralization, greenhouse gas emissions, nitrogen transformation kinetics, and microbial community regulation. Finally, we discuss methodological limitations and future research needs, with emphasis on long-term field observations, environmentally relevant exposure conditions, and multi-factor coupling experiments.

Because microplastics occur in multiple environmental compartments, their biogeochemical effects must be interpreted in an ecosystem-specific context. In aquatic and marine environments, hydrodynamic transport, buoyancy, aggregation with organic and inorganic particles, biofouling, settling, and resuspension control particle residence time and transfer to sediments. In terrestrial soils, retention, accumulation, soil aggregation, pore-structure alteration, water retention, and rhizosphere interactions are more important. In sediments, redox stratification, burial, sediment–water exchange, and bioturbation regulate organic matter mineralization, nitrogen transformation, and greenhouse gas fluxes. This review therefore distinguishes among terrestrial, aquatic, marine, and sedimentary settings when discussing microplastic effects on carbon and nitrogen cycling [3,4,5].

## 2. Physicochemical Evolution and Composite Pollution Characteristics of Microplastics

### 2.1. Chemical Diversity of Polymer Types

The chemical composition of microplastics forms the basis of their environmental behavior. Traditional plastics such as polyethylene, polypropylene, polystyrene, and polyvinyl chloride consist primarily of hydrocarbon chains, exhibit extremely low bioavailability, and are regarded as inert carbon pools. Polyamides (nylon), containing nitrogen due to amide bonds, undergo carbon–nitrogen coupling processes in the environment. Biodegradable plastics such as polylactic acid (PLA) and polybutylene terephthalate (PBT) contain ester bonds, making them susceptible to microbial enzymatic degradation; their carbon release rates are significantly higher than those of conventional plastics [6].

The chemical diversity of microplastics is also reflected in the composition of additives in different polymers. Functional additives such as plasticizers, flame retardants, antioxidants, and colorants are incorporated into plastic products during production. These substances leach out as microplastics degrade, potentially exerting toxicity on microorganisms or serving as potential carbon sources [7,8]. The types and concentrations of additives vary depending on the polymer type, production batch, and intended use, resulting in nearly infinite possible combinations of chemical compositions for microplastics.

### 2.2. Physical Diversity of Size and Morphology

The size distribution of microplastics spans three orders of magnitude (1 μm to 5 mm), and microplastics within different size ranges exhibit distinctly different environmental behaviors. Smaller microplastics have a higher specific surface area, allowing them to adsorb more microorganisms, enzymes, and nutrients, while also being more easily ingested by organisms or transported across cell membranes [9,10]. There is an interaction between size effects and polymer type: small-sized (25–38 μm) polylactic acid microplastics significantly inhibit plant nitrogen uptake, whereas medium-sized (600–700 μm) polyethylene microplastics actually enhance nitrogen availability by promoting nitrification [11].

Particle size also determines the capacity of microplastics to serve as microbial attachment surfaces. Smaller particles provide more attachment sites for microbial adhesion, extracellular polymeric substance accumulation, and biofilm development, while larger fragments and fibers may provide more stable surfaces and modify pore networks. Plastic-associated biofilms, often called the plastisphere, can differ from surrounding soil, sediment, or water microbial communities and may enrich microorganisms involved in organic carbon degradation, nitrogen fixation, nitrification, denitrification, or methanogenesis. Thus, particle size affects not only transport and toxicity but also the potential of microplastics to act as microhabitats for functional microorganisms [12,13].

Experimental evidence supports the role of microplastics as microbial microhabitats. Microscopic observations have demonstrated biofilm development on plastic surfaces, while sequencing and metagenomic analyses have shown that plastisphere communities differ from those in surrounding water, soil, sediment, and natural substrates. These plastic-associated communities may contain microorganisms and functional pathways involved in organic carbon degradation and nitrogen transformation. More direct functional evidence has been obtained from coastal salt-marsh sediment microcosms exposed to PE, PVC, polyurethane foam, and PLA microplastics, in which the abundance of the ammonia-oxidation gene amoA increased by approximately 210% and denitrification rates increased by approximately 2–3.2-fold. These responses were accompanied by changes in microbial community composition and functional gene expression. Collectively, these findings indicate that microplastics can provide distinct colonization surfaces and physicochemical niches that influence both microbial community assembly and biogeochemical functions [14,15].

Nanometric plastic particles require particular attention because their mechanisms of action may differ from those of micrometric particles. Their high surface-area-to-volume ratio, colloidal behavior, and mobility increase the probability of direct interactions with microbial membranes, extracellular enzymes, and intracellular components. Nanoplastics may cross biological barriers, induce oxidative stress, disrupt membrane integrity, and interfere with microbial metabolism. They should therefore not be regarded simply as smaller microplastics but as particles with distinct physicochemical and toxicological behavior [2,10].

Microplastics exhibit diverse morphologies, including fibrous, fragmentary, microbead, and film-like forms. Fibrous microplastics primarily originate from textile washing and fishing net wear; they constitute a high proportion of environmental microplastics and readily form network structures, potentially altering water and air movement as well as element diffusion by entangling plant roots or clogging soil pores [3,16]. Fragment-shaped microplastics have sharp edges and may physically damage cell membranes; microbeads are typically regular spheres, commonly found in personal care products, and their environmental behavior is more similar to that of colloidal particles [4].

### 2.3. Environmental Transformation During the Degradation Process

After release into the environment, microplastics undergo physical, chemical, and biological aging that progressively alters their size, surface chemistry, and ecological behavior. Mechanical abrasion generates smaller fragments, including nanoscale particles. Ultraviolet radiation promotes photooxidation and polymer-chain scission, whereas microbial extracellular enzymes contribute to biodegradation. Although biodegradable plastics degrade faster than conventional polymers, degradation in natural environments is still controlled by temperature, moisture, oxygen availability, microbial activity, and exposure time [17,18].

Aging-induced surface oxidation is particularly important because it increases the environmental reactivity of microplastics. Photooxidation, abrasion, and biodegradation introduce oxygen-containing functional groups, including carbonyl, hydroxyl, and carboxyl groups, and generate cracks and rough surfaces. These changes increase surface polarity, hydrophilicity, charge density, and the number of adsorption sites. Consequently, aged microplastics generally interact more strongly with heavy metals and polar organic contaminants through electrostatic attraction, surface complexation, ligand exchange, and hydrogen bonding. Roughened surfaces also promote microbial attachment and biofilm formation [13,17,19].

**Figure 1:**
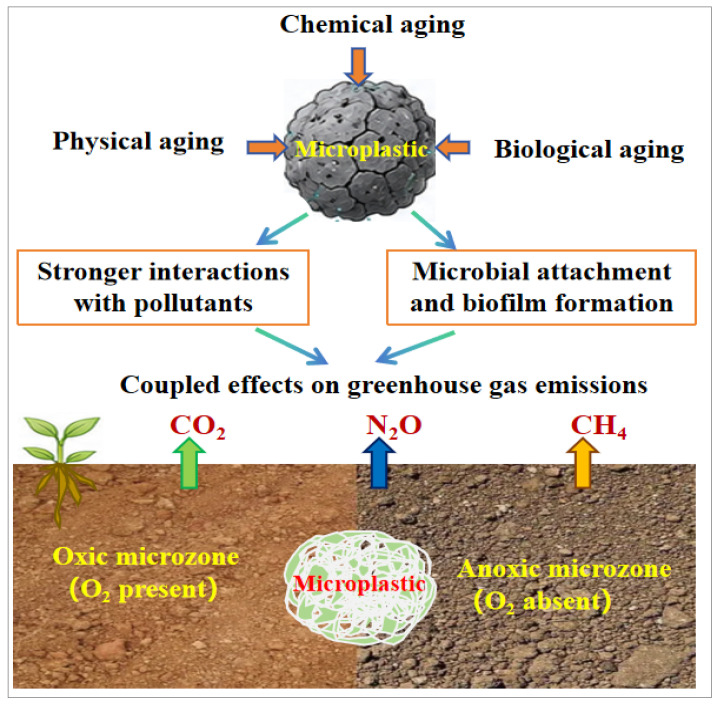
Schematic diagram of environmental aging processes of microplastics and their driving mechanisms on greenhouse gas emissions.

Compared with fresh particles, aged microplastics may influence greenhouse gas emissions more strongly through coupled physical, chemical, and microbial pathways (Figure 1). Increased roughness and surface area facilitate colonization by microbial biofilms; oxidation and fragmentation enhance reactivity and spatial dispersion; and dissolved organic carbon or low-molecular-weight compounds released during aging can serve as microbial substrates. These changes may stimulate CO_2_ production, promote incomplete denitrification and N_2_O accumulation under oxygen-limited conditions, and enhance CH_4_ production in anaerobic microsites. The magnitude and direction of these effects depend on polymer type, aging pathway, redox status, and microbial community composition [2,17,20].

### 2.4. Complex Systems of Co-Occurring Contaminants

**Figure 2:**
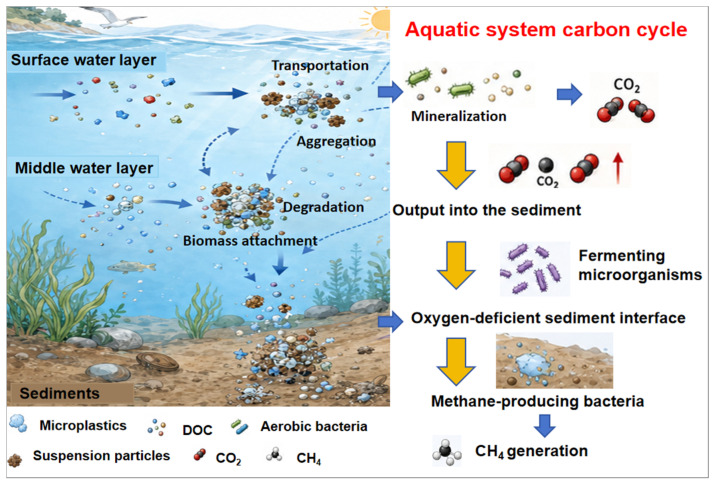
Mechanism diagram of microplastics regulating the transformation and fate of organic carbon in aquatic systems.

Microplastics do not exist in isolation in the environment but form complex pollution systems with heavy metals, persistent organic pollutants, antibiotics, and other substances (Figure 2). The high specific surface area and hydrophobicity of microplastics make them carriers for pollutants: polyvinyl chloride (PVC) microplastics can adsorb heavy metals such as copper, cadmium, and lead; polystyrene microplastics accumulate polycyclic aromatic hydrocarbons (PAHs) and polybrominated diphenyl ethers (PBDEs); and polyamide microplastics exhibit strong adsorption capacity for antibiotics [21]. Pollutant binding occurs through surface adsorption, pore diffusion, hydrophobic partitioning, and chemical complexation, and is regulated by polymer type, aging state, contaminant properties, and environmental conditions [19].

Microplastic-heavy metal interactions are particularly relevant to nitrogen cycling because denitrifying microorganisms are sensitive to metal exposure (Figure 2). Aged particles can concentrate metals within the plastisphere, increasing local exposure of denitrifiers. At elevated concentrations, metals may disrupt membrane integrity, inhibit electron transport, and suppress enzymes involved in nitrate, nitrite, nitric oxide, and nitrous oxide reduction. Inhibition of nitrous oxide reductase may favor incomplete denitrification and increase N_2_O accumulation. However, adsorption onto microplastics may also reduce freely dissolved metal concentrations in the surrounding medium. Microplastics may therefore enhance or buffer metal toxicity depending on metal speciation, particle aging state, and environmental conditions [19,21,22].

When organisms ingest contaminant-loaded microplastics, associated pollutants may be released, transformed, or transferred along food webs, producing combined toxic effects and indirectly affecting metabolic activity and elemental transformation functions [23]. These interactions indicate that the biogeochemical consequences of microplastics cannot be evaluated solely from polymer properties; they also depend on co-contaminants and their bioavailability.

## 3. Effect Mechanisms of Microplastics on the Carbon Cycle

### 3.1. Microplastics as a Direct Input to the Carbon Pool

Microplastics themselves are high-carbon polymers, and their input directly increases the total organic carbon content in the environment. Studies show that in coastal wetland sediments, the carbon contribution of microplastics can range from 0.001% to 1.197% [24]. Biodegradable microplastics, such as polylactic acid (PLA), release large amounts of dissolved organic carbon in a short period due to the easy hydrolysis of their ester bonds, serving as an easily utilizable carbon source for microorganisms. In contrast, conventional microplastics, such as polyethylene (PE), release carbon at a slower rate but can still gradually release low-molecular-weight organic compounds through the aging process, providing a continuous carbon source for microorganisms [6].

### 3.2. Regulation of Organic Carbon Mineralization by Microplastics

Microplastics regulate organic carbon dynamics through physical and chemical pathways that differ among soils, sediments, and aquatic systems. Physical effects include changes in aggregate stability, pore-size distribution, pore connectivity, water retention, aeration, and diffusion of oxygen and solutes. Chemical effects include direct polymer-derived carbon input, release of dissolved organic carbon, additive leaching, and adsorption or desorption of contaminants. The response of organic carbon mineralization therefore reflects the combined action of matrix restructuring, substrate supply, contaminant exposure, and microbial metabolism [5,9,25,26].

In soils, microplastics can alter aggregate formation and pore architecture, thereby changing microbial access to protected organic matter. Fibers may entangle fine particles to form artificial aggregates, whereas film-like particles may disrupt particle contact and reduce aggregate stability [3]. Because soil aggregates physically protect organic carbon, their disruption can expose previously protected carbon to microbial decomposition and increase CO_2_ emissions [27]. Changes in pore connectivity also regulate water and gas movement. Reduced permeability can create anaerobic microsites, shifting carbon transformation from aerobic respiration toward methanogenesis [20,26].

In sediments and aquatic systems, different controls dominate. In sediments, microplastics may influence organic matter mineralization by altering particle deposition, permeability, benthic bioturbation, and exchange across the sediment–water interface. Reduced bioturbation can limit mass exchange and lower mineralization rates or CO_2_ fluxes [5]. In water columns, transport, aggregation, biofouling, and sinking determine whether plastic-associated organic matter remains suspended, is degraded in the water column, or is transferred to sediments. Thus, the same polymer or particle size may produce different carbon-cycle responses depending on the environmental compartment [4,13].

Microplastic-induced shifts in microbial communities can further mediate organic carbon mineralization. Enrichment of copiotrophic bacteria and rapidly growing decomposers may accelerate the utilization of labile dissolved organic carbon, whereas changes in fungal decomposers can modify the breakdown of structurally complex organic matter. Under oxygen-limited conditions, enrichment of fermentative microorganisms and methanogens may redirect carbon flow from aerobic CO_2_ production toward CH_4_ formation. Consequently, changes in carbon mineralization reflect not only physical disturbance and substrate release but also shifts in microbial functional composition and metabolic pathways [8,20,25].

### 3.3. The Promoting Effect of Microplastics on Greenhouse Gas Emissions

The impact of microplastics on greenhouse gas emissions, such as carbon dioxide and methane, has been confirmed by numerous studies. The addition of polyethylene microplastics typically promotes soil respiration and increases carbon dioxide emissions, which is related to their ability to increase soil porosity, improve aeration conditions, and stimulate microbial activity [28]. Soluble organic carbon released from biodegradable microplastics can act as an electron donor, further promoting microbial metabolism and carbon dioxide production [25].

**Figure 3:**
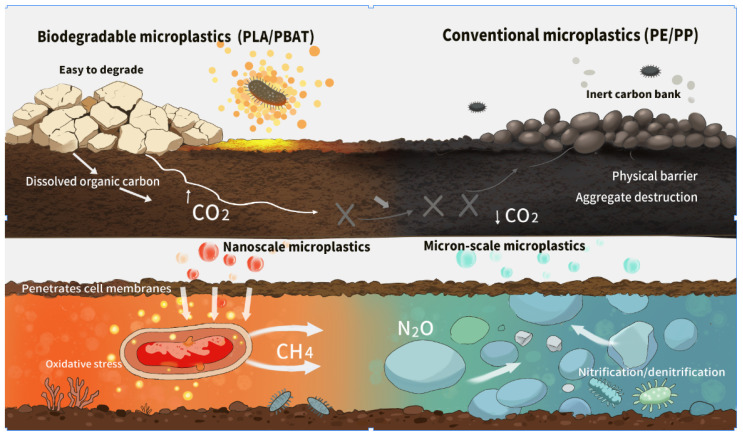
Mechanisms by which the physicochemical characteristics of microplastics influence organic carbon mineralization and greenhouse gas emissions. Arrows indicate increases or decreases in the corresponding processes, while X-shaped symbols denote degraded microplastics.

Regarding methane, the effects of microplastics are mixed. When polyethylene coexists with hydrocarbons, it can significantly increase the gene abundance of methanogenic bacteria and promote methane emissions [20]. In contrast, the co-application of polyethylene terephthalate and biochar may inhibit methane production by increasing the soil redox potential [29]. The degree of microplastic degradation is also critical; the abundant oxygen-containing functional groups on the surface of degraded microplastics may enhance their stimulating effect on greenhouse gas emissions [2]. These studies indicate that the regulatory role of microplastics on greenhouse gas emissions depends on their type, coexisting substances, and the redox state of the environment [30] (Figure 3).

Biodegradable and conventional microplastics affect greenhouse gas emissions through different dominant mechanisms. Biodegradable microplastics such as PLA release relatively labile dissolved organic carbon during hydrolysis and microbial degradation (Figure 3). This additional carbon can stimulate heterotrophic respiration and increase CO_2_ production; under oxygen-limited conditions, it can also provide electron donors for denitrification and methanogenesis, potentially increasing N_2_O and CH_4_ emissions. Conventional polymers such as PE and PP release less bioavailable carbon over short timescales and more often affect greenhouse gases through physical changes in aeration, water retention, pore connectivity, and plastic-surface colonization. Thus, biodegradable microplastics mainly exert substrate-driven chemical effects, whereas conventional microplastics more often act through physical disturbance and long-term aging [16,25,31].

The effects of microplastics on N_2_O and CH_4_ should therefore be interpreted as redox- and community-dependent. Enhanced aeration may suppress CH_4_ production but promote nitrification-related N_2_O formation. Conversely, reduced oxygen diffusion or anaerobic microsite formation may stimulate denitrification and methanogenesis. The final greenhouse gas response depends on polymer type, degradation state, soil or sediment moisture, redox conditions, and the abundance of nitrifying, denitrifying, and methanogenic microorganisms [20,30,32].

## 4. Mechanisms of Microplastics’ Impact on the Nitrogen Cycle

### 4.1. Differential Regulation of Nitrogen Transformation Processes

The nitrogen cycle consists of a series of microorganism-driven redox reactions, including nitrogen fixation, ammonification, nitrification, denitrification, and anammox. The effects of microplastics on these processes exhibit complex regulatory patterns (Table 1).

Impact on Nitrification: Nitrification is the key step in the conversion of ammonium nitrogen to nitrate via nitrite, carried out synergistically by ammonia-oxidizing bacteria, ammonia-oxidizing archaea, and nitrite-oxidizing bacteria. Studies report that microplastics generally reduce the relative abundance of nitrifying and ammonia-oxidizing bacteria and inhibit nitrification activity [28]. The inhibitory mechanisms may involve the toxicity of additives released by microplastics, microenvironmental anoxia, and alterations in microbial community structure [5]. However, there are exceptions: medium-sized polyethylene microplastics promote nitrification by improving soil aeration, thereby providing plants with more nitrate nitrogen [11].

Effects on denitrification: Denitrification gradually reduces nitrate to gaseous nitrogen (nitric oxide, nitrous oxide, and nitrogen gas) and is the primary pathway for nitrogen export from ecosystems. The effects of microplastics on denitrification are bidirectional and depend on carbon bioavailability, oxygen conditions, contaminant exposure, and particle concentration. Labile dissolved organic carbon released from biodegradable or aged microplastics may serve as an electron donor for denitrification [15]. In addition, anoxic microsites induced by microplastics may favor the enrichment of denitrifying microorganisms and increase N_2_O production [32]. However, excessively high concentrations of microplastics may inhibit denitrifying enzyme activity through toxic effects [22].

Effects on nitrogen fixation: Nitrogen fixation converts atmospheric nitrogen into biologically available ammonia and is mediated by symbiotic and free-living nitrogen-fixing microorganisms. Some studies have reported an increased relative abundance of nitrogen-fixing microorganisms in microplastic-amended environments, possibly because plastic surfaces provide distinct colonization niches. These findings suggest that microplastics may alter the potential for biological nitrogen fixation; however, increases in the relative abundance of nitrogen-fixing microorganisms do not necessarily indicate higher in situ nitrogen-fixation rates [12,28].

**Figure 4:**
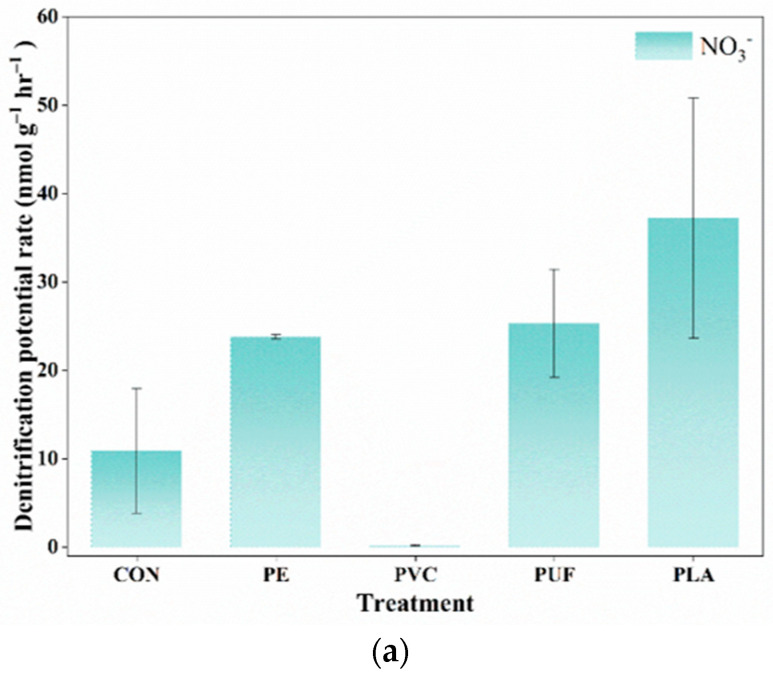

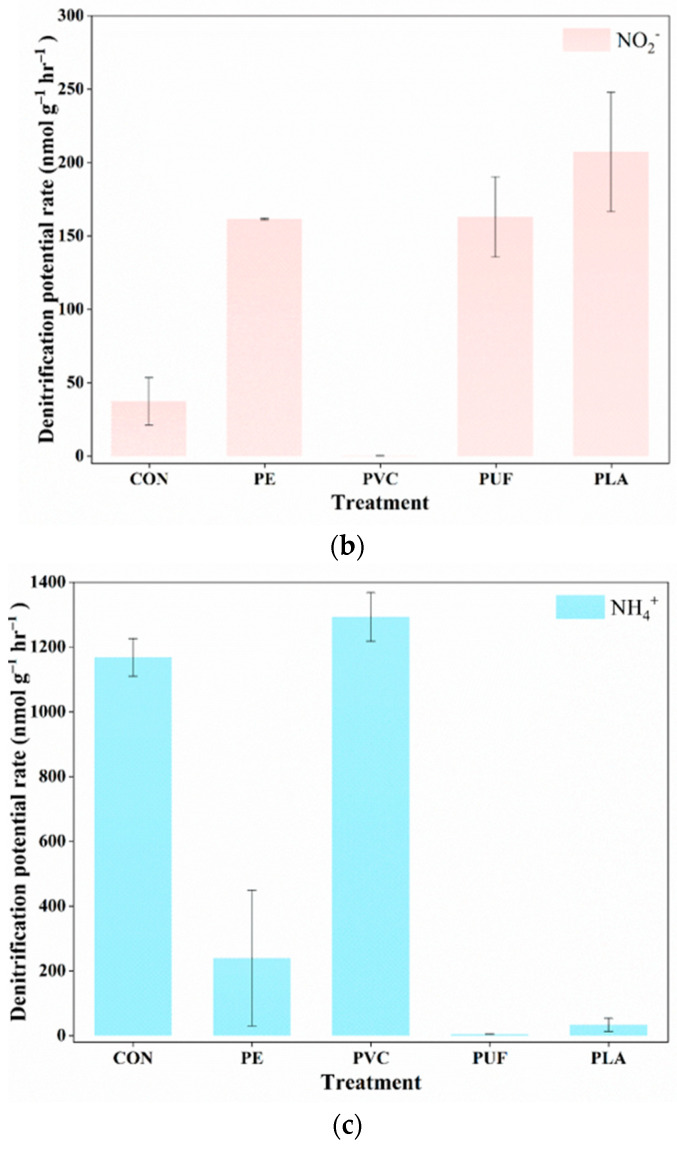
Effects of different microplastic polymers on dissolved inorganic nitrogen concentrations in salt-marsh sediment microcosms after 16 days of incubation. (**a**) Nitrate (NO_3_^−^) concentrations; (**b**) Nitrite (NO_2_^−^) concentrations; (**c**) Ammonium (NH_4_^+^) concentration. Values are means ± SE (*n* = 3).

Polymer-specific effects on nitrogen cycling have also been observed in sedimentary systems. In salt-marsh sediment microcosms, PE, PVC, polyurethane foam (PUF), and PLA produced contrasting changes in NO_3_^−^, NO_2_^−^, and NH_4_^+^ concentrations after 16 days of incubation (Figure 4). These patterns indicate polymer-dependent changes in dissolved inorganic nitrogen availability [14].

These nitrogen-cycle responses are closely linked to shifts in microbial community structure and functional groups. Microplastics can alter the abundance of nitrifiers, denitrifiers, nitrogen-fixing microorganisms, and organisms involved in nitrogen immobilization by changing habitat structure, oxygen availability, substrate supply, contaminant exposure, and plastisphere formation. Enrichment of denitrifiers in oxygen-limited microsites may increase N_2_O production, whereas suppression of nitrifiers may reduce nitrate availability. Microplastic-induced changes in nitrogen cycling should therefore be understood as both physicochemical and microbial community-mediated responses [12,28,32].

The influence of microplastics on nitrogen transformation is also ecosystem-specific. In soils, retention and accumulation modify nitrification and denitrification by changing aeration, water retention, rhizosphere structure, and plant–microbe competition for nitrogen. In aquatic and marine systems, particle mobility, suspended biofilms, and hydrodynamic transport determine the distribution of plastisphere microorganisms and their contribution to nitrogen transformation in the water column. In sediments, microplastics can alter redox gradients, sediment–water exchange, and the spatial coupling between nitrification and denitrification. Effects on nitrogen fixation, nitrification, denitrification, and N_2_O production should therefore be evaluated within clearly defined environmental settings [3,4,5].

### 4.2. Coupling Effects of Size and Type

The interaction between polymer type and particle size has a particularly significant impact on the nitrogen cycle. A systematic study by Zhang Jinbo’s team at Hainan University revealed the differential effects of conventional polyethylene and biodegradable polylactic acid on the “soil nitrogen transformation–plant nitrogen uptake” coupling relationship at different particle sizes [11].

For polyethylene microplastics, plants exhibited the highest nitrogen uptake rates at medium sizes (600–700 μm). Mechanistic analysis indicated that nitrification rates were higher at this size, providing plants with more nitrate nitrogen. Larger sizes may fail to sufficiently stimulate microbial activity due to insufficient specific surface area, while smaller sizes may cause slight inhibition due to the release of additives or alterations in the microenvironment.

For polylactic acid (PLA) microplastics, small sizes (25–38 μm) significantly inhibited plant nitrogen uptake rates. The study found that the nitrogen mineralization rate at this size was 6 to 11 times higher than that of other sizes; however, the rate of ammonium ion fixation was far higher than the mineralization rate. This, in turn, prolonged the retention time of ammonium ions in the soil, inhibiting direct plant uptake of ammonium ions. This finding overturns the simplistic expectation that “enhanced mineralization necessarily promotes nitrogen supply” and reveals the dynamic changes in the microbe-plant competitive relationship.

The coupled effects of size and type stem from the chemical differences between the two types of polymers. Polyethylene is an inert hydrocarbon that primarily influences the nitrogen cycle through physical interactions; polylactic acid can be rapidly utilized by microorganisms, and its carbon release alters microbial metabolic activity and elemental requirements, thereby regulating nitrogen transformation processes through stoichiometric mechanisms [11].

### 4.3. Rebalancing of Competition Between Plants and Microorganisms

These polymer and size-dependent changes in nitrogen transformation can also rebalance competition between plants and microorganisms for available nitrogen. This shift in competitive balance is regulated by multiple factors. The type of microplastic determines the bioavailability of carbon sources, and the rapid turnover of degradable plastics exacerbates microbial nitrogen demand. Particle size influences the spatial relationship between microplastics, plant roots, and microorganisms; smaller particles are more likely to enter the rhizosphere microenvironment and directly interfere with root-microbe interactions. Specific functional bacteria (e.g., nitrogen-fixing and phosphorus-solubilizing bacteria) enriched in the biofilm on microplastic surfaces may alter rhizosphere nutrient transformation dynamics, thereby indirectly modulating competitive relationships [28].

**Figure 5:**
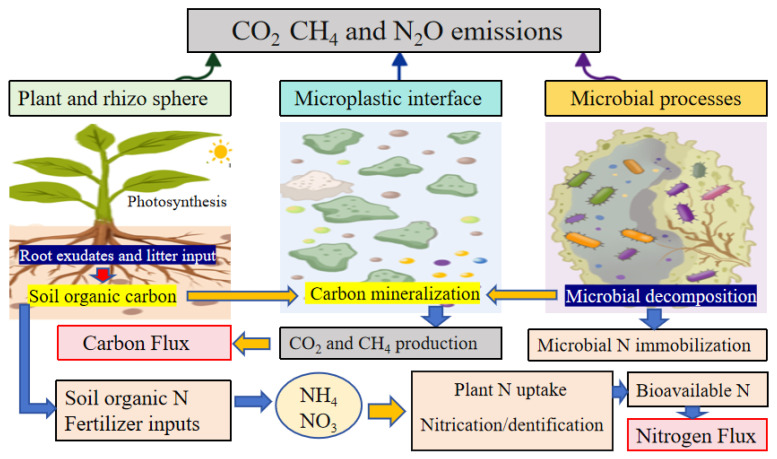
Microplastic-driven C–N coupling and plant–microbe competition regulate soil GHG emissions.

From an ecosystem perspective, microplastic-induced inhibition of plant nitrogen uptake may reduce primary productivity and litterfall input, leading to long-term feedback on the carbon–nitrogen coupling cycle (Figure 5). This chain reaction warrants particular attention in agricultural ecosystems, as it directly impacts crop yields and fertilizer use efficiency [11].

## 5. Research Challenges and Methodological Limitations

Current understanding of how microplastics influence carbon and nitrogen cycles primarily stems from laboratory simulation studies. While such studies offer the advantages of high controllability and in-depth mechanistic analysis, their limitations are becoming increasingly apparent. Laboratory studies typically use fresh, unweathered commercial microplastics at concentrations far higher than actual environmental levels (usually 1% to 5% by mass, whereas microplastic concentrations in environmental soil typically range from 0.01% to 0.1% by mass). Exposure times are often limited to weeks or months, making it difficult to reflect long-term cumulative effects [28].

The complexity of environmental conditions is difficult to replicate in the laboratory. In the natural environment, microplastics undergo long-term aging, resulting in surface properties that are markedly different from those of fresh particles; microplastics coexist with various pollutants in the environment, leading to synergistic or antagonistic effects; climatic factors (temperature fluctuations, alternating wet and dry conditions, freeze–thaw cycles) and biological factors (plant growth, animal disturbance) interact to influence the behavior and effects of microplastics [2]. Conclusions derived from simplified laboratory treatments may be amplified, attenuated, or even reversed under field conditions.

The widespread use of non-aged commercial microplastics is a particular limitation. Such particles usually lack oxygen-containing functional groups, surface cracks, irregular morphologies, adsorbed contaminants, and established microbial communities. As a result, they may underestimate aging-related adsorption, microbial colonization, and dissolved organic carbon release, while overestimating effects related to uniform particle shape, high purity, or artificially high concentration. Future experiments should include environmentally aged particles, realistic concentration gradients, mixed polymer types, irregular morphologies, longer exposure periods, and field validation [17,28].

Another limitation is that many simulation studies do not clearly distinguish among terrestrial, freshwater, marine, and sedimentary exposure scenarios. Static soil incubations do not represent hydrodynamic transport in rivers, estuaries, or coastal waters, whereas water-column experiments may not capture long-term retention, aggregation, and rhizosphere interactions in soils. Sediment experiments require explicit consideration of vertical redox gradients, burial, bioturbation, and sediment–water exchange. Future studies should define the target environmental compartment and use system-specific experimental conditions, exposure concentrations, particle aging states, and functional endpoints [3,4,5].

## 6. Future Research Directions

### 6.1. Long-Term In Situ Studies

It is imperative to shift the research paradigm from short-term simulations to long-term in situ observations. Long-term monitoring stations for microplastic pollution must be established across different ecosystem types (farmland, forests, wetlands, freshwater, and marine environments) to systematically track the physicochemical evolution of microplastics, the succession of plastic communities, changes in carbon and nitrogen fluxes, and their responses to climatic factors [2].

In situ studies should focus particularly on microplastic accumulation hotspots: long-term plastic-mulched farmland, landfills, contaminated soil in industrial parks, riverine sediment zones, deep-sea sedimentary areas, and polar ice cores. These areas have high microplastic loads and long residence times, serving as natural laboratories for observing long-term effects [33]. These areas have high microplastic loads and long residence times, serving as natural laboratories for observing long-term effects [2]. At the same time, standardized methods should be developed to ensure data comparability across different studies, including sampling methods, microplastic extraction and identification, and the determination of functional indicators [34].

### 6.2. Application of Systems Biology Techniques

Systems biology techniques provide powerful tools for in-depth analysis of the interaction mechanisms among microplastics, microorganisms, and elemental cycles. Metagenomics can comprehensively reveal the composition and functional gene potential of microbial communities in plastic-contaminated environments; metatranscriptomics and metaproteomics reflect real-time dynamics of gene expression; and metabolomics captures changes in small-molecule metabolites, linking genotype to phenotype [2].

Stable isotope probing is a key method for tracking elemental fluxes. Stable isotope-labeled microplastics can trace the pathways of plastic carbon conversion into microbial biomass, metabolites, and greenhouse gases; isotope-labeled nitrogen sources (ammonium, nitrate, nitrogen gas) can quantify nitrogen transformation rates and fate under the influence of microplastics [2]. Combining nano-secondary ion mass spectrometry with isotope labeling allows for the observation of element flux at single-cell resolution, revealing metabolic activity at the microscopic scale.

### 6.3. Life-Cycle Assessment of Biodegradable Plastics

The widespread adoption of biodegradable plastics is altering the composition of microplastic pollution. Compared to conventional plastics, biodegradable plastics generally undergo faster carbon turnover and may exert more immediate effects on carbon and nitrogen transformations than conventional plastics [11]. There is an urgent need to conduct life-cycle assessments of biodegradable plastics to systematically compare their advantages and disadvantages with alternative materials (conventional plastics, paper, reusable products) in terms of resource consumption, carbon emissions, and ecological impacts.

The assessment should pay particular attention to the environmental fate and ecological effects of degradation products. Under conditions favorable for degradation, rapid mineralization may increase short-term carbon dioxide release. Intermediate degradation products may exhibit ecological toxicity distinct from that of the parent polymers, while the degradation process may consume oxygen, release organic acids, and alter local environmental conditions [25,31]. These factors should be incorporated into the ecological risk assessment framework for biodegradable plastics.

Dissolved organic carbon released from biodegradable plastics may also contribute to eutrophication-related processes in aquatic ecosystems. In nutrient-rich waters, labile plastic-derived carbon can stimulate heterotrophic microbial growth and respiration, accelerate oxygen consumption, and promote hypoxia. Enhanced microbial activity may also alter nitrogen and phosphorus regeneration and change algal–bacterial interactions. However, this pathway depends on nutrient availability, hydrodynamic residence time, oxygen status, degradation rate, and microbial community composition, and should be tested through coupled measurements of dissolved organic carbon release, oxygen dynamics, nutrient fluxes, microbial succession, and algal responses [26,31].

## 7. Conclusions

The effects of microplastics on carbon and nitrogen cycles are not uniform. Available evidence indicates that microplastics influence elemental cycling through interacting pathways, including direct carbon input, alteration of soil and sediment physical structure, release of dissolved organic matter and additives, modification of redox conditions, contaminant interactions, and restructuring of microbial communities. The direction and magnitude of these effects depend on polymer type, particle size, morphology, aging state, environmental matrix, exposure concentration, and co-contaminants. Microplastics should therefore be considered a heterogeneous group of materials with context-dependent biogeochemical effects rather than a single pollutant class with fixed ecological functions.

The distinction between conventional and biodegradable microplastics is particularly important. Conventional polymers such as PE and PP mainly affect carbon and nitrogen cycling through physical disturbance, surface colonization, and long-term aging. Biodegradable polymers may release more bioavailable carbon and more directly alter microbial metabolism, nitrogen immobilization, mineralization, and greenhouse gas production. Their apparent environmental advantages should therefore be evaluated cautiously, because rapid degradation may reduce visible plastic accumulation while accelerating carbon release and modifying nitrogen availability.

Current knowledge remains limited by short-term laboratory studies, frequent use of fresh commercial particles, high exposure concentrations, and insufficient separation of ecosystem-specific mechanisms. These limitations hinder extrapolation to long-term field conditions. Future studies should integrate environmentally aged particles, realistic concentration gradients, long-term in situ monitoring, stable isotope tracing, multi-omics approaches, and standardized analytical protocols. Such work is needed to distinguish generalizable mechanisms from system-specific responses and improve predictions of microplastic effects on carbon–nitrogen coupling under real environmental conditions.

In summary, incorporating microplastic diversity and ecosystem specificity into carbon and nitrogen cycle research is essential for accurate assessment of plastic pollution risks. Rather than providing a final resolution to this emerging problem, this review highlights mechanistic complexity, current uncertainties, and research priorities for understanding microplastic-mediated changes in biogeochemical cycles.

## Figures and Tables

**Table 1:** Summary of the Effects of Microplastic Diversity on Key Carbon and Nitrogen Cycle Processes.

Properties of Microplastics	Organic Carbon Mineralization	CO_2_	CH_4_	Nitrification	Denitrification	Plant Nitrogen Uptake
Conventional microplastics	↓/—	↑	↑/—	↑/↓	↑	—/↑
Biodegradable microplastics	↑↑	↑↑	↑	↓	↑	↓(small size)
Nano-scale	↑	↑↑	↑	↓↓	↑	↓↓
Micron-scale	—/↑	↑	—	↑/↓	↑	—
Aged microplastics	↑	↑↑	↑↑	↓	↑↑	↓

Note. “↑” indicates promotion, “↓” indicates inhibition, “—” indicates no effect, “↑↑/↓↓” indicates a strong effect.

## Data Availability

No new data were created or analyzed in this study. Data sharing is not applicable to this article.
